# Erythroid Differentiation Regulator 1 as a Novel Biomarker for Hair Loss Disorders

**DOI:** 10.3390/ijms18020316

**Published:** 2017-02-03

**Authors:** Yu Ri Woo, Sewon Hwang, Seo Won Jeong, Dae Ho Cho, Hyun Jeong Park

**Affiliations:** 1 Department of Dermatology, Yeouido St. Mary’s Hospital, College of Medicine, The Catholic University of Korea, Seoul 07345, Korea; w1206@naver.com (Y.R.W.); dingting@naver.com (S.H.); 2 Institute of Clinical Medical Research, Yeouido St. Mary’s Hostpital, College of Medicine, The Catholic University of Korea, Seoul 07345, Korea; cmcos@hanmail.net; 3 Department of Life Science, Sookmyung Women’s University, Seoul 04310, Korea; cdhkor@sookmyung.ac.kr

**Keywords:** hair loss, alopecia, erythroid differentiation regulator 1, inflammation

## Abstract

Erythroid differentiation regulator 1 (Erdr1) is known to be involved in the inflammatory process via regulating the immune system in many cutaneous disorders, such as psoriasis and rosacea. However, the role of Erdr1 in various hair loss disorders remains unclear. The aim of this study was to investigate the putative role of Erdr1 in alopecias. Skin samples from 21 patients with hair loss disorders and five control subjects were retrieved, in order to assess their expression levels of Erdr1. Results revealed that expression of Erdr1 was significantly downregulated in the epidermis and hair follicles of patients with hair loss disorders, when compared to that in the control group. In particular, the expression of Erdr1 was significantly decreased in patients with alopecia areata. We propose that Erdr1 downregulation might be involved in the pathogenesis of hair loss, and could be considered as a novel biomarker for hair loss disorders.

## 1. Introduction

Hair loss is a heterogeneous disease entity, characterized by diverse clinical presentations which range from only a solitary hair loss patch, to the complete loss of body hair in the hair-bearing areas [1]. Hair loss can be classified as non-scarring or scarring alopecia, based on the presence of follicular ostia, which are the openings of the hair follicles through which hairs emerge from the skin. Scarring alopecia is characterized by the lack of follicular ostia; and the condition is irreversible [2]. By contrast, a common form of non-scarring alopecia, alopecia areata (AA), is associated with autoimmune attacks on hair follicles, characterized by the presence of follicular ostia [3]. The progression of AA is diverse, and it fluctuates over time [3]. Another hair loss disorder is traction alopecia, which is caused by secondary traumatic tensile forces to the hair [4]. 

A wide range of hypotheses exist regarding the etiology and pathogenesis of hair loss disorders. Most of these disorders have abnormalities in hair follicle cycling, with modifications in hair follicle morphology [5]. Moreover, diverse molecules are suspected to be involved in the complex pathogenesis of hair loss. For example, autoimmune inflammation, characterized by T-cell predominant infiltrates and associated cytokines such as interferon (IFN)-γ, interleukin (IL)-2, IL-12, IL-18, and tumor necrosis factor (TNF)-α, have been found to be associated with the pathogenesis of AA [3,6]. In addition, an imbalance between pro-inflammatory and anti-inflammatory cytokines is suspected to be related to persistent alopecic lesions [7]. Kim et al. have reported that stress-related peptides such as corticotrophin-releasing hormones, adrenocorticotropic hormones, and α-melanocyte-stimulating hormones, are elevated in AA, implying that stress can initiate and aggravate AA [8]. However, the exact contributory stimuli and triggers of hair loss have not yet been elucidated [9]. 

IL-18, a member of the IL-1 superfamily, acts as a functional pro-inflammatory cytokine, and induces the production of IFN-γ [10]. IL-18 has been implicated in the pathophysiology of diverse chronic inflammatory disorders, such as psoriasis, lupus erythematosus, atopic dermatitis, and rosacea, by stimulating T helper 1 responses [11,12,13]. In hair loss disorders, the single-nucleotide polymorphisms in IL18 have been implicated in the susceptibility of Koreans to AA [14]. In addition, Lee et al. [15] have reported that patients with AA have abnormal serum IL-18 levels. In our previous study, we have observed negative correlations between IL-18 and erythroid differentiation regulator 1 (Erdr1) in human keratinocytes [16]. Recently, it has been reported that Erdr1 plays an important role in the inflammatory process via regulating the immune system in various cutaneous chronic inflammatory disorders, such as psoriasis and rosacea [16,17]. 

However, to the best of our knowledge, no study has reported the expression of Erdr1 in hair follicles. In addition, the precise role of Erdr1 in diverse hair loss disorders remains unknown. Therefore, the aim of this study was to analyze the expression patterns of Erdr1 in normal hair follicles and diverse types of alopecias. For this purpose, we compared Erdr1 expression levels in human scalp specimens of healthy controls and patients with various hair loss disorders. Investigating the molecular factors associated with hair loss disorders is important for understanding their pathogenesis.

## 2. Results

### 2.1. Erythroid Differentiation Regulator 1 (Erdr1) Is Expressed by Normal Hair Follicular Epithelium

**Figure 1:**
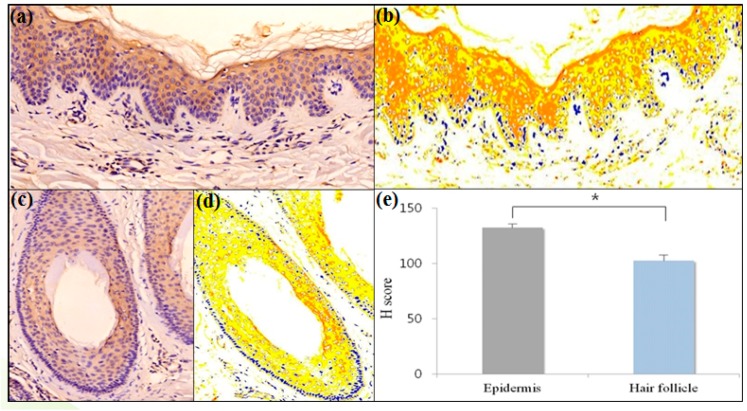
(**a**) Erythroid differentiation regulator 1 (Erdr1) expression in scalp epidermis of a healthy control; Immunostaining of Erdr1 is shown in brownish-red color; (**b**) HistoQuant™ measurement for the intensity of Erdr1 expression in scalp epidermis of the healthy control; Red—strong positive; orange—moderately positive; yellow—weakly positive; blue—negative nuclei; white—negative pixels in normal epidermis; (**c**) Erdr1 expression in the hair follicle of a healthy control; (**d**) HistoQuant™ measurement for the intensity of Erdr1 expression in hair follicle of the healthy control; (**e**) The intensities of Erdr1 expression in the epidermis and hair follicles of the healthy control were quantitatively evaluated with an H score using HistoQuant™; Erdr1 immunostaining, original magnification (**a**–**d**) ×200; * indicates a *p*-value less than 0.05.

To determine whether Erdr1 is expressed in the hair follicles, immunohistochemical staining of Erdr1 in healthy controls (*n* = 5) was performed. The expression of Erdr1 was observed in the epidermis and hair follicle skin samples of normal, healthy controls (Figure 1a,c). The intensity of Erdr1 expression was quantitatively analyzed using the HistoQuant™ (3DHISTECH Ltd., Budapest, Hungary) digital analysis system. Results are shown in Figure 1b,d. The ratios of weak, moderate, and strong positive pixels to total pixels of Erdr1, are summarized in Table 1. The mean H scores for Erdr1 expression in the epidermis and hair follicles were 132.24 ± 3.15 and 102.45 ± 5.68, respectively. The expression level of Erdr1 in the hair follicles was significantly (*p* = 0.008) lower than that in the epidermis of healthy controls (Figure 1e, Table 1). 

**Figure 2:**
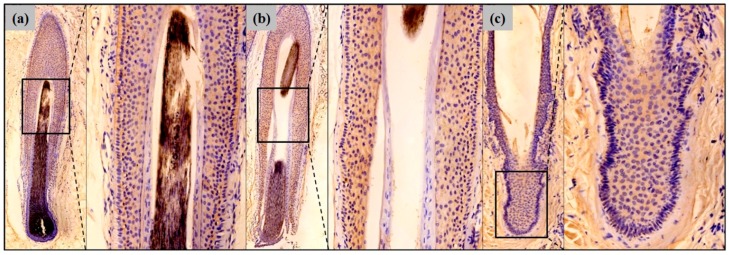
Erdr1 expression in a healthy scalp hair follicle; (**a**) Erdr1 expression in the anagen phase of the follicle; A high-power image of Erdr1-positive cells in the outer root sheath of the follicle is shown; (**b**) Healthy hair follicle in the catagen phase; A high-power image of Erdr1-positive cells in the outer root sheath of the catagen hair follicle is shown; (**c**) Erdr1 expression in the telogen phase; Erdr1 immunostaining, original magnification (**a**–**c**) ×50; inserts ×200.

To further determine the differences in Erdr1 expression, based on hair follicle cycling, each hair follicle was classified by its cycle. As shown in Figure 2, Erdr1 was expressed in the anagen, catagen, and telogen phases of follicles. Regarding the expression density, Erdr1 was more intensely expressed in the catagen follicle (mean ± SEM = 112.62 ± 6.12), than in either the anagen (mean ± SEM = 104.31 ± 8.14) or the telogen follicle (mean ± SEM = 96.62 ± 5.43). In particular, the outer root sheaths of anagen and catagen follicles showed higher expression levels of Erdr1 than the inner root sheath (Figure 2), although the difference in Erdr1 expression observed throughout the overall hair follicle cycling, was not statistically significant (*p* = 0.210).

### 2.2. Downregulation of Erdr1 in Alopecia Areata 

**Figure 3:**
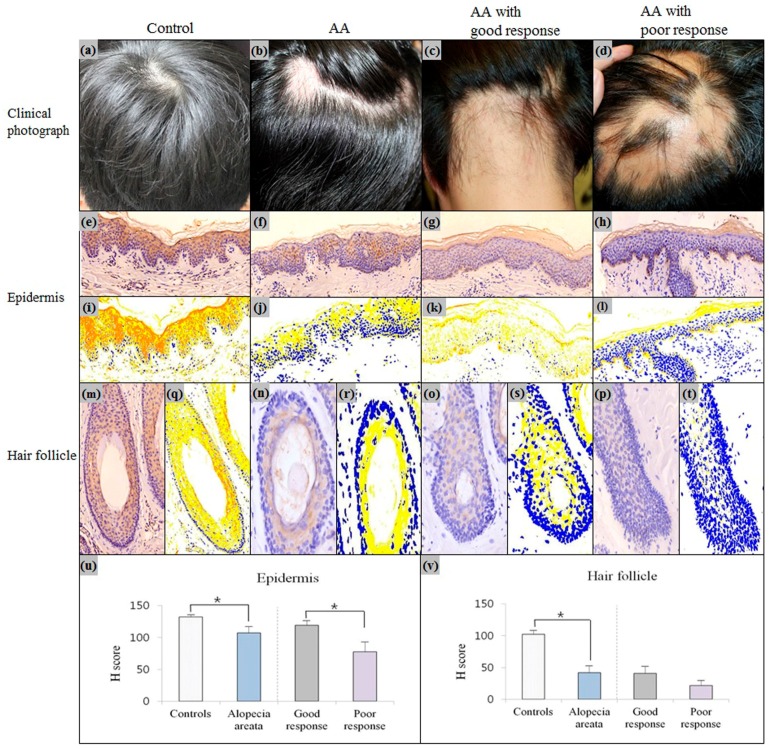
(**a**–**d**) Clinical photograph of a healthy control, alopecia areata, alopecia areata with good clinical response, and alopecia areata with poor clinical response; (**e**–**h**) Erdr1 staining patterns of epidermis in a healthy control, alopecia areata, alopecia areata with good clinical response, and alopecia areata with poor clinical response. (**i**–**l**) HistoQuant™ measurement for the intensity of Erdr1 expression in the epidermis of a healthy control, alopecia areata, alopecia areata with good clinical response, and alopecia areata with poor clinical response; Red—strong positive; orange—moderately positive; yellow—weakly positive; blue—negative nuclei; white—negative pixels; (**m**–**p**) Erdr1 staining patterns of the hair follicle in a healthy control, alopecia areata, alopecia areata with good clinical response, and alopecia areata with poor clinical response; (**q**–**t**) HistoQuant™ measurement for the intensity of Erdr1 expression in the hair follicle of a healthy control, alopecia areata, alopecia areata with good clinical response, and alopecia areata with poor clinical response; (**u**) The intensity of Erdr1 expression in the epidermis in alopecia areata was quantitatively evaluated using the HistoQuant™ H score; (**v**) Erdr1 expression in the hair follicles of alopecia areata was quantitatively compared to each group using the H score; Erdr1 immunostaining, original magnification (**e**–**i**) ×200; (**m**–**t**) ×400. AA: alopecia areata. * indicates a *p*-value less than 0.05.

Compared to healthy controls, expression levels of Erdr1 in the epidermis (*p* = 0.048) and hair follicles (*p* = 0.004) of AA patients, were significantly decreased (Figure 3). To further investigate the role of Erdr1 in AA, the clinical status of patients with AA was assessed.

To treat AA, an intralesional injection with triamcinolone acetonide (8 mg/mL) was given to the individual once every two weeks, or immunotherapy with diphenylcyclopropenone was performed once every week. The concentrations of diphenylcyclopropenone were gradually increased, as follows: 0.0001%, 0.001%, 0.01%, and 0.1%. An intralesional corticosteroid injection was used in patients with less than 50% scalp involvement. For patients with more than 50% scalp involvement, topical immunotherapy with diphenylcyclopropenone was applied [18]. AA patients were divided into either a good or poor response group, according to the treatment’s response. A good response was defined when 50% or greater hair growth was achieved for the involved areas after six months of treatment, whereas a poor response was defined when less than 50% hair growth was achieved for the involved areas after six months of treatment [19,20]. The intensity (H score) of Erdr1 expression was quantitatively calculated using the HistoQuant™ digital analysis system. The mean H score of Erdr1 expression in the epidermis of the poor response group was significantly lower (mean ± SEM = 77.74 ± 15.42) than that of the good response group (mean ± SEM = 119.20 ± 11.06; *p* = 0.043) (Figure 3u). The mean H score for Erdr1 expression in the hair follicles of the poor response group (mean ± SEM = 21.56 ± 8.13) was also lower than that of the good response group (mean ± SEM = 40.80 ± 11.54), although the difference was not statistically significant (*p* = 0.41) (Figure 3v). 

When the severity of alopecia was classified as mild (three or fewer alopecic patches), moderate (more than three alopecic patches or a patch greater than 3 cm), or severe (alopecia totalis or alopecia universalis) [21], the severity of AA was not significantly correlated with the intensity of Erdr1 expression (*p* = 0.34).

As part of the study, serum hemoglobin and ferritin levels in AA patients were measured, and their correlation with the Erdr1 expression level was determined. The mean serum hemoglobin level was 15.05 ± 2.19 g/dL (reference: 12–16 g/dL). The mean serum ferritin level was 115.42 ± 25.43 ng/mL (reference: 11–306 ng/mL). These mean serum levels in AA patients were within normal ranges. There was no significant correlation between the serum hemoglobin level and Erdr1 expression in the epidermis (Spearman’s correlation coefficient, rho = 0.682, *p* = 0.136), or hair follicles (Spearman’s correlation coefficient, rho = 0.316, *p* = 0.542). Similarly, there was no significant correlation between the serum ferritin level and Erdr1 expression in the epidermis (Spearman’s correlation coefficient, rho = 0.371, *p* = 0.468), or hair follicles (Spearman’s correlation coefficient, rho = 0.429, *p* = 0.397). However, of the nine patients with AA, one (11%) had an extremely low serum ferritin level (5.7 ng/mL), with Erdr1 downregulation (epidermis: H score = 79.39; hair follicle: H score = 36.63).

### 2.3. Erdr1 Downregulation in Scarring Alopecia

**Figure 4:**
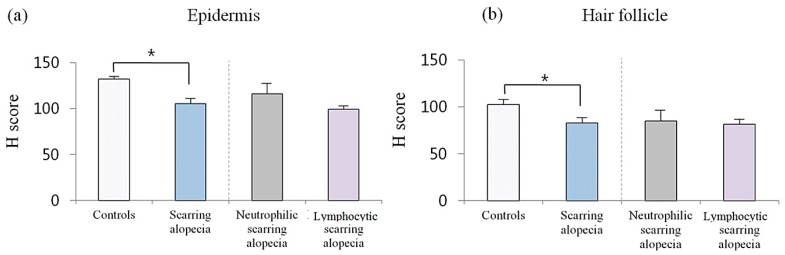
Erdr1 expression in scarring alopecia. The intensity of Erdr1 expression in scarring alopecia was quantitatively evaluated using the HistoQuant™ H score; (**a**) The mean H score for Erdr1 expression in the epidermis; (**b**) Erdr1 expression levels in hair follicles of patients with scarring alopecia. * indicates a *p*-value less than 0.05.

The intensity of Erdr1 expression levels in patients with scarring alopecia (*n* = 8) were analyzed and expressed as H scores. The mean H score of Erdr1 expression in the epidermis was significantly (*p* = 0.011) decreased in scarring alopecia (mean ± SEM = 105.60 ± 5.29), when compared to that of healthy controls (mean ± SEM = 132.24 ± 3.15). The mean H score of Erdr1 expression in the hair follicles was also significantly (*p* = 0.04) decreased in scarring alopecia (mean ± SEM = 82.87 ± 5.32), when compared to that in healthy controls (mean ± SEM = 102.45 ± 5.68) (Figure 4). 

**Figure 5:**
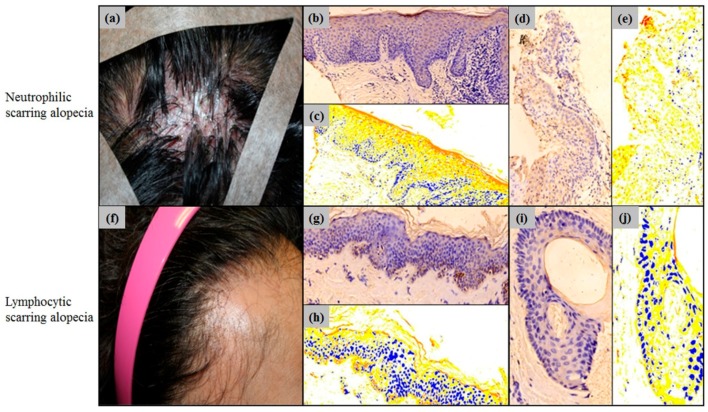
(**a**) Representative clinical photograph of neutrophilic scarring alopecia showing folliculitis decalvans; (**b**) Erdr1 staining patterns of the epidermis in neutrophilic scarring alopecia; (**c**) HistoQuant™ measurement for the intensity of Erdr1 expression; Red—strong positive; orange—moderately positive; yellow—weakly positive; blue—negative nuclei; white—negative pixels; (**d**) Erdr1 staining patterns of the hair follicle in neutrophilic scarring alopecia; (**e**) HistoQuant™ measurement for the intensity of Erdr1 expression in the hair follicle of neutrophilic scarring alopecia; (**f**) Representative clinical photograph of lymphocytic scarring alopecia showing frontal fibrosing alopecia; (**g**) Erdr1 staining patterns of the epidermis in lymphocytic scarring alopecia; (**h**) HistoQuant™ measured the intensity of epidermal Erdr1 expression in lymphocytic scarring alopecia; (**i**) Erdr1 staining patterns of the hair follicle; (**j**) HistoQuant™ measured the intensity of Erdr1 expression in the hair follicle; Erdr1 immunostaining, original magnification (**b**–**e**,**g**,**h**) ×200; (**i**,**j**) ×400.

The specimens with scarring alopecia were sub-classified into neutrophilic (*n* = 3) and lymphocytic (*n* = 5) scarring alopecia, and were analysed for Erdr1 expression (Figure 5). The epidermal Erdr1 expression was more decreased in neutrophilic scarring alopecia, than the lymphocytic scarring alopecia. In addition, the follicular Erdr1 expression was slightly decreased in neurtrophilic scarring alopecia, when compared to the lymphocytic scarring alopecia (Figure 5).

To quantitatively analyze the intensity of Erdr1 expression, HistoQuant™ was used to measure the H score. The mean H score for epidermal Erdr1 expression in lymphocytic scarring alopecia (mean ± SEM = 99.31 ± 4.06) was lower than that in the neutrophilic scarring alopecia group (mean ± SEM = 116.07 ± 10.88). Follicular Erdr1 expression was also lower in lymphocytic scarring alopecia (mean ± SEM = 81.67 ± 5.64), when compared to that in neutrophilic scarring alopecia (mean ± SEM = 85.24 ± 13.29). However, no significant (*p* > 0.05) difference in Erdr1 expression in the epidermis or hair follicles was found between lymphocytic and neutrophilic scarring alopecia groups (Figure 4).

### 2.4. Epidermal Erdr1 Expression Was Downregulated in Traction Alopecia 

**Figure 6:**
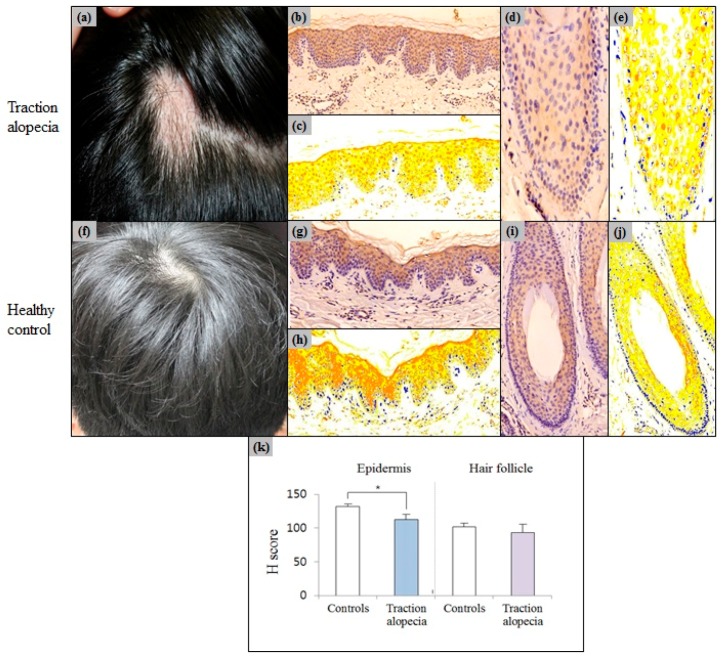
(**a**) Clinical photograph of traction alopecia; (**b**) Erdr1 staining patterns of the epidermis in traction alopecia; (**c**) HistoQuant™ measurement for the intensity of epidermal Erdr1 expression in traction alopecia patients; (**d**) Erdr1 staining patterns of the hair follicle in traction alopecia; (**e**) HistoQuant™ measured the intensity of Erdr1 expression in the hair follicles; (**f**) Clinical photograph of a healthy control; (**g**) Erdr1 staining patterns of the epidermis in healthy control; (**h**) HistoQuant™ measurement for the intensity of epidermal Erdr1 expression in the healthy control; (**i**) Erdr1 staining patterns of the hair follicle from the healthy control; (**j**) HistoQuant™ measurement for the intensity of Erdr1 expression in hair follicles; (**k**) The intensity of Erdr1 expression in traction alopecia was quantitatively determined using the HistoQuant™ H score and compared to that in healthy controls; Erdr1 immunostaining, original magnification (**b**,**c**,**g**,**h**) ×200; (**d**,**e**,**i**,**j**) ×400; * indicates a *p*-value less than 0.05.

Erdr1 expression levels in patients with traction alopecia (*n* = 4) were compared to those in healthy controls. The intensity of epidermal Erdr1 expression in patients with traction alopecia (mean ± SEM = 113.99 ± 7.46), was significantly (*p* = 0.032) lower than that in the control group (mean ± SEM = 132.24 ± 3.15). However, the expression intensity of Erdr1 in traction alopecia hair follicles (mean ± SEM = 94.76 ± 12.60), was similar (*p* = 0.98) to that of the control group (mean ± SEM = 102.45 ± 5.68) (Figure 6). 

### 2.5. Comparison of Erdr1 Expression in Alopecia Subgroups

**Figure 7:**
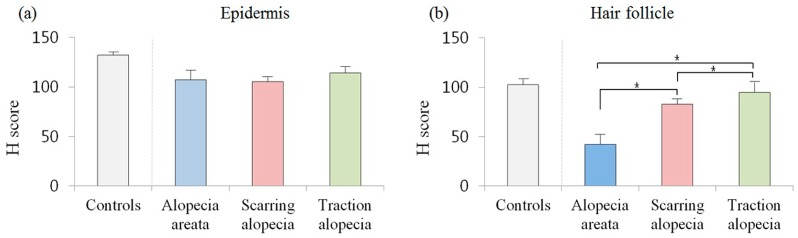
Comparison of Erdr1 expression in alopecia subgroups; The intensities of Erdr1 expression levels in alopecia areata, scarring alopecia, and traction alopecia were quantitatively evaluated using the HistoQuant™ H score; (**a**) The mean H score for Erdr1 expression in the epidermis of various hair loss disorders; (**b**) Erdr1 expression in the hair follicles of various hair loss disorders. * indicates a *p*-value less than 0.05.

Next, the expression levels of Erdr1 in different alopecia subgroups were determined. In the epidermis of alopecia subgroups, Erdr1 expression levels were significantly downregulated (AA: mean ± SEM, 107.36 ± 9.77; scarring alopecia: mean ± SEM, 105.60 ± 5.29; traction alopecia: mean ± SEM, 113.99 ± 7.46), when compared to that in healthy controls (mean ± SEM, 132.24 ± 3.15; *p* = 0.023). However, there was no significant (*p* = 0.468) difference in the epidermal expression of Erdr1 among different alopecia subgroups. By contrast, a significant (*p* = 0.001) difference of Erdr1 expression in hair follicles was found among the different alopecia subgroups. Follicular Erdr1 expression in AA (mean ± SEM, 42.16 ± 10.76) was significantly downregulated, when compared to that in other subgroups (scarring alopecia, mean ± SEM: 82.87 ± 5.32, *p* = 0.002; traction alopecia, mean ± SEM: 94.76 ± 12.60, *p* = 0.003) (Figure 7). 

## 3. Discussion

Although enormous datasets are available in hair biology, little is known about the pathological mechanisms of various hair loss disorders. Therefore, we examined the role of the novel molecule Erdr1 in these disorders. Our results demonstrated that Erdr1 was downregulated in diverse hair loss disorders, including AA, scarring alopecia, and traction alopecia. In particular, Erdr1 was significantly downregulated in hair follicles of AA patients, when compared to that in hair follicles of patients with scarring alopecia and traction alopecia. 

It has been reported that Erdr1 is expressed in erythroleukaemia cell lines and various tissues, including the thymus, placenta, brain, intestine, and bone marrow [22]. In a previous study, we found that Erdr1 is expressed in human epidermis, sebaceous glands, eccrine glands, vessels, and nerves [23]. However, the expression of Erdr1 in a healthy hair’s follicular structure has not been yet reported. In the present study, we demonstrated that Erdr1 was expressed in the hair follicle, although the intensity of its expression was lower in follicles, than that in the epidermis. Erdr1 is a regulator of cell homeostasis, by regulating cell survival and apoptosis [24]. At low concentrations, Erdr1 can enhance cell survival. However, it induces cell apoptosis at high concentrations [24]. Because Erdr1 acts differently, depending on its concentrations, such a difference in the expression between healthy epidermis and hair follicles, might contribute to divergent mechanisms that regulate cell homeostasis. 

In this study, Erdr1 downregulation was found in both scarring alopecia and AA, consistent with results of previous studies, which show Erdr1 downregulation in chronic inflammatory disorders, such as psoriasis and rosacea [17,25]. This suggests that inflammation is related to Erdr1 downregulation in hair loss disorders. In a variety of hair loss disorders, cell-mediated follicular inflammation is a major histopathological finding, suggesting the principal role of cell-mediated immunity in these disorders [26]. Although the mechanisms that underlie the destruction of follicular stem cells in scarring alopecia have not been completely understood, it has been proposed that damages to the bulge area, due to follicular inflammation, can result in an aberrant hair cycle, consequently causing scarring of hair follicles. In AA, the collapse of the hair follicle’s immune privilege and peribulbar inflammatory cells infiltrates, have been considered as major pathogenic factors [27]. Moreover, T helper 1 cytokines in AA are implicated in the dysregulation of the hair follicle’s immune homeostasis [9]. Erdr1 is negatively regulated by IL-18 [16], an IFN-γ inducing molecule. Therefore, we proposed that Erdr1 downregulation might be involved in the inflammatory cascade in AA. Significant downregulation of follicular Erdr1 expression in AA might be explained by these points. Traction alopecia is generally not considered to be accompanied by inflammatory infiltrates in the hair follicle [28]. However, epidermal downregulation of Erdr1 was also observed in traction alopecia. Traction alopecia might occur after traumatic damage. Mild inflammation after the trauma might have affected the expression of Erdr1 in the epidermis. 

A variety of etiological factors have been suspected as contributory factors in the pathogenesis of AA, including drugs, nutritional deficiencies, and hormones [21]. In addition, a number of studies have confirmed a possible association between AA and stress [8,29]. Erdr1 functions as a stress-related survival factor. It is expressed in cells undergoing stressful conditions [22], suggesting a potential positive relationship between Erdr1 and the initiation of AA. However, our data showed that Erdr1 expression was somewhat downregulated in AA. We suspect that the cell-mediated inflammation associated with Erdr1 might play a more important role in the etiology of AA than stress-related biology. Additional studies are needed to clarify the possible regulatory mechanisms of Erdr1 involved in AA. 

In addition to the above-mentioned factors, anemia has been implicated in hair loss disorders. However, the present study found no relationship between iron deficiency anemia and AA. Boffa et al. [20] have reported that serum levels of ferritin and hemoglobin are within normal limits in patients with AA, in agreement with our results. In addition, we observed no correlation between Erdr1 and serologic markers, including hemoglobin and ferritin levels. However, Erdr1 expression was significantly downregulated in one patient, who also had critically low ferritin. Thus, the possible relationship between the two cannot be discounted. Moreover, anemia and iron deficiency have been implicated as risk factors for certain hair loss disorders, including AA, androgenic alopecia, and telogen effluvium [30,31,32]. Although the exact mechanisms by which iron deficiency and iron deficiency anemia affect hair loss remain unclear, some authors have suspected that hair follicles are the most actively dividing cells in humans, and that they might be sensitive to even slight decreases in iron availability [30,32]. In addition, Erdr1 is known to be able to stimulate hemoglobin synthesis in both human and murine erythroid leukaemia cell lines [22]. Its decreased expression might decrease hemoglobin synthesis and its iron-binding capacity. Therefore, decreased hemoglobin-inducing capacity, caused by Erdr1 downregulation, might have a direct effect on hair-bearing tissues, without necessarily altering systemic levels, consequently resulting in hair loss.

In the present study, Erdr1 expression was found in both healthy epidermis and healthy hair follicles. In addition, Erdr1 was downregulated in the epidermis and hair follicles of various hair loss disorders. The formation of hair follicles is affected by reciprocal epithelial and mesenchymal interactions [5]. These interactions involve different molecules, such as epithelial Wnt ligand and β-catenin, required for regenerating hair follicles [33,34]. Research on these associated molecules is currently in progress. Preliminary findings suggest that Erdr1 might be a novel molecule involved in this interaction. However, more studies are needed to confirm such an association.

This study has some limitations. First, the sample size of patients with different types of alopecia was small. Thus, studies with larger samples and various types of alopecia are needed. In addition, more clinical laboratory parameters might be needed to further confirm this association.

To date, a variety of alopecia biomarkers have been discovered and validated for AA [8,18,35,36,37]. However, their exact pathogenic roles have not yet been fully studied. In the present study, decreased Erdr1 expression was observed in hair loss disorders, including AA, scarring alopecia, and traction alopecia. Epidermal and follicular downregulation of Erdr1 was observed in scarring alopecia and AA. In particular, significant downregulation of follicular Erdr1 was observed in AA, when compared to that in scarring alopecia. However, epidermal Erdr1 downregulation, not follicular Erdr1 downregulation, was found in traction alopecia. Based on these differences in the expression pattern of Erdr1, depending on disease entities, the level of Erdr1 might be used as a marker to diagnose hair loss disorders, along with clinicopathologic characteristics (Table 2). Significant downregulation of epidermal Erdr1 was especially observed in the poor response group of AA, when compared to that in the good response group. Therapeutic response in AA is difficult to predict, even when clincopathological correlation is used. Therefore, Erdr1 expression might have potential as a useful tool for predicting AA prognosis. Additional studies are needed to determine the possible relationships between Erdr1, inflammatory cascades, altered stress-related pathways, and hemoglobin-inducing capacity in hair loss disorders. 

In conclusion, results revealed that Erdr1 expression was downregulated in alopecia. Our results suggest that Erdr1 is a novel molecule involved in the pathogenesis of various hair loss disorders.

## 4. Materials and Methods

### 4.1. Subjects

Clinical charts of patients with hair loss disorders who visited the outpatient dermatology department at Yeouido St. Mary’s Hospital from September 2013 to April 2016, were retrospectively reviewed in this study. Among them, specimens from 21 patients with hair loss disorders who had taken skin biopsies were retrieved. For comparison, scalp skin samples were taken from five healthy subjects. Patient demographics are summarized in Table 3. Among available records, data of AA patients who had received laboratory tests, including serum hemoglobin and ferritin measurements, were collected. This study was approved by the Ethics Committee of the Catholic University of Korea (SC16SISI0105). It was conducted according to the principles of the Declaration of Helsinki. 

### 4.2. Immunohistochemical Analyses

For immunohistochemical staining with the anti-Erdr1 antibody, formalin-fixed skin tissues embedded in paraffin were cut into serial sections, with a thickness of 6 µm. Formalin-fixed skin tissues embedded in paraffin were deparaffinized in xylene, and rehydrated in alcohol. These slides were incubated with hydrogen peroxide to eliminate endogenous peroxidases. Slides were then incubated with hydrogen peroxide to eliminate endogenous peroxidases. To retrieve antigens, a citrate buffer (10 nM, pH = 6) was used to autoclave slides under standard conditions for 10 min. A polygonal anti-Erdr1 antibody was produced in rabbit, immunized with a full-length recombinant His-tagged Erdr1 protein. Anti-Erdr1 antibody (diluted 1:1000) was used as a primary antibody. It was incubated with slides overnight at 4 °C in a wet chamber. To detect the antigen, a streptavidin-biotin-peroxidase detection system (Cap-plus Detection Kit; Invitrogen, Carlsbad, CA, USA) was used. 3-amino-9-ethylcarbazole was used as the chromogen. As a negative control, normal rabbit immunoglobulin G (IgG) (Zymed Laboratories, San Francisco, CA, USA) was used. 

### 4.3. Quantitative Digital Analysis for Erdr1 Expression

To quantitatively measure the level of Erdr1 expression, a Panoramic Scan™ slide scanner (3D HISTECH, Budapest, Hungary) was used. The degree of expression was assessed with the DensitoQuant application component of Pannoramic Viewer software™ (3D HISTECH). Before analysis, sample RGB values for cells were manually set. DensitoQuant™ (3DHISTECH Ltd., Budapest, Hungary) was then used to automatically assess the intensity of immunostained-positive cells, and presented them in different coloured images based on the expression intensity, as follows: weak positive (yellow), moderate positive (orange), strong positive (red), negative nuclei (blue), and negative pixels (white). Based on different colour intensities, the H (‘histological’) scores were then calculated using the following equation: H score = 1 × (% cells 1+) + 2 × (% cells 2+) + 3 × (% cells 3+); 0 = negative staining, 1+ = weak staining, 2+ = moderate staining, 3+ = strong staining [28].

### 4.4. Statistics

IBM SPSS version 21.0 (SPSS Inc., Chicago, IL, USA) was used for all statistical analyses. Means and the standard error of means (SEM) were used to determine baseline characteristics of the study population. Statistical analyses were performed with Mann-Whitney and Spearman’s rank tests. Statistical significance was considered when the *p*-value was less than 0.05.

## Figures and Tables

**Table 1:** Erdr1 expression in a healthy scalp.

Ratio of Positive Immunoreactivity	Epithelium	Hair Follicle	*p*-Value
Ratio of negative pixels to total pixels	7.76 ± 2.15	13.57 ± 3.81	0.310
Ratio of weak-positive pixels to total pixels	53.65 ± 3.92	70.60 ± 3.24	0.032
Ratio of moderate-positive pixels to total pixels	37.16 ± 2.50	15.65 ± 2.66	0.008
Ratio of strong-positive pixels to total pixels	1.43 ± 0.39	0.18 ± 0.05	0.008
H score	132.24 ± 3.15	102.45 ± 5.68	0.008

Data are means ± standard error of mean (SEM); H-score = 1 × (% of weakly positive pixels) + 2 × (% of moderately positive pixels) + 3 × (% of strongly positive pixels).

**Table 2:** Proposed diagnostic clues for various hair loss disorders.

Disorders	Clinical Findings	Histopathology	Associated Biomarkers
**Alopecia areata**	- Smooth oval or round patch on the hair bearing area	- Peribulbar lymphocytic infiltration of affected follicles - Increased numbers of miniaturized follicles	Inflammation; ↓ Erdr1 in the epidermis; ↓ Erdr1 in the HF; ↑ IFN-γ, CXCL10, CXCL9, IL-13, CCL18, CCL26, TSLP [35] around the HF; stress; ↑ CRH, α-MSH, ACTH [8] in the epidermis and HF; neuromediators; ↓ VIP receptor [36] in the HF; ↑ Substance P [37] in the perifollicular nerve fibers; keratin; ↓ KRT83–86, KRT35, KRT81, KRT40, KRT75, KRTAP1 [34] in the HF
**Scarring alopecia**	- Permanent loss of hair follicle manifested by absence of follicular ostia	- Destruction of hair follicle	Inflammation; ↓ Erdr1 in the epidermis; ↓ Erdr1 in the HF
**Traction alopecia**	- History for the application of tensile force to the hair follicle	- Absence of the inflammatory infiltration around hair follicle - Marked damage of inner root sheath	Inflammation; ↓ Erdr1 in the epidermis

Upward arrow indicates increased expression of certain protein or gene; Downward arrow indicates decreased expression of certain protein or gene; The numbers in parentheses refer to references numbers; ACTH: adrenocorticotropic hormone; α-MSH: alpha-melanocyte-stimulating hormone; CCL: CC-chemokine ligand; CRH: corticotropin-releasing hormone; CXCL: CXC-chemokine ligand; Erdr1: Erythroid differentiation regulator 1; HF: hair follicle; IFN-γ: Interferon-gamma; KRT: keratin; KRTAP1: keratin-associated protein 1; TARC: thymus and activation-regulated chemokine; TSLP: thymic stromal lymphopoietin; VIP: vasoactive intestinal peptide.

**Table 3:** Demographic data of the study population.

Characteristics	Alopecia (*n* = 21)	Normal Controls (*n* = 5)
Age (range)	32 (12–68)	43 (25–60)
Male/Female	12/9	2/3
Types of alopecia	21	-
Alopecia areata	9 (42.6)	-
Scarring alopecia	8 (38.1)	-
Lymphocytic	5	-
Neutrophilic	3	-
Traction alopecia	4 (19.3)	-

Data are presented as *n* (%), unless otherwise indicated.
